# Protein kinase activity is associated with CD63 in melanoma cells

**DOI:** 10.1186/1479-5876-3-42

**Published:** 2005-11-30

**Authors:** Joji Iida, Amy PN Skubitz, James B McCarthy, Keith M Skubitz

**Affiliations:** 1 Department of Laboratory Medicine and Pathology, The University of Minnesota Medical School, Minneapolis, MN 55455, USA; 2 Department of Medicine, The University of Minnesota Medical School and the Masonic Cancer Center, Minneapolis, MN 55455, USA

## Abstract

**Background:**

The tetraspan protein CD63, originally described as a stage-specific melanoma antigen but also present in a number of normal cells, regulates melanoma cell growth in nude mice, motility in serum containing media, and adhesion to several extracellular matrix proteins. CD63 has been reported to associate with β1 and β2 integrins, but the mechanism of signal transduction by CD63 is not clear. This study examined whether CD63 is associated with protein kinase and can transmit signals in melanoma cells.

**Methods:**

Immunoprecipitation and radiolabeling were used to test for association of protein kinase activity with CD63. Adhesion of cells to monoclonal antibodies immobilized to microtiter plates was used to examine the ability of CD63 to transmit signals.

**Results:**

CD63 was capable of transmitting a signal in melanoma cells that required extracellular calcium. In the absence of extracellular calcium at the time of binding to the CD63 mAb, the cell was no longer responsive to stimulation by CD63. Immunoprecipitation studies demonstrated protein kinase activity associated with CD63, and phosphoamino acid analysis revealed that most of this protein kinase activity was due to serine kinase activity.

**Conclusion:**

The current study suggests that serine protein kinase activity associated with CD63 may play a role in signaling by CD63 in melanoma cells.

## Background

CD63 was initially described as the ME491 antigen of melanoma cells [1-8]. CD63 is not expressed in normal melanocytes, but is expressed in nevi and many melanoma cells. CD63 is also expressed in a number of normal cells [1-5,9-18]. A number of studies have suggested that CD63 may regulate melanoma cell functions. Transfection with CD63 of NIH-3T3 cells transformed with H-ras, resulted in cells with lower growth rates following subcutaneous injection in nude mice [19]. Transfection of the CD63 negative melanoma cell line KM3 with genomic CD63 resulted in cells with similar in vitro growth rates as control cells, but much slower growth rates in vivo when cells were injected intradermally in nude mice [20]. In addition, intravenous injection of CD63 transfected KM3 cells resulted in fewer peritoneal and subcutaneous metastases [20]. These data, therefore, suggest that CD63 may regulate in vivo growth and metastatic capability [20]. Finally, transfection of the highly motile KM3 cells with CD63 resulted in suppression of in vitro motility in serum-containing media that was potentiated by CD63 mAbs, and increased adhesion and migration on fibronectin, laminin, and collagen [21]. In contrast, transfection of antisense CD63 cDNA into melanoma cells endogenously expressing CD63, resulted in increased cell motility and invasiveness in vitro[22]. Thus, CD63 appears capable of regulating melanoma cell functions, although the mechanism of this regulation is unclear.

CD63 is a member of the tetraspan family, traversing the membrane four times and having a major extracellular loop and a small extracellular loop, with short intracellular amino and carboxy termini [1,2,4,5,14,18,23-25]. The intracellular domains of CD63 are small and have no clear motif known to be involved in signal transmission.

In this study, the properties of the adhesion of melanoma cells to immobilized CD63 mAb suggested that CD63 was capable of transmitting a signal that requires extracellular calcium. Protein kinase activity, predominantly in the form of serine kinase activity, was found to be associated with CD63 in these cells. The association of serine protein kinase activity with CD63 may play a role in signaling by CD63 in melanoma.

## Methods

### Cell Culture

Highly metastatic human melanoma cells, A375SM, which were selected by in vivo experimental metastasis assays of parent A375P cells in nude mice [26] were kindly provided by Dr. I. J. Fidler (M.D. Anderson Hospital Cancer Center, Houston, TX). The cells were maintained in Eagle's Minimum Essential Medium (EMEM) supplemented with 10% FBS, vitamin solution, 50 mg/ml gentamycin, and 1 mM sodium pyruvate. Cells were routinely used after fewer than 15 passages from frozen stocks in order to minimize phenotypic drift.

### Monoclonal antibodies (mAbs)

The CD63 mAbs, AHN-16 (IgG2a), AHN-16.1 (IgG1), AHN-16.2 (IgG2b), AHN-16.3 (IgG_2b_), and AHN-16.5 (IgG_1_) were previously described [15]. mAb U131 (IgG1) was previously described [27]. mAb 9.2.27 that recognizes the core protein of melanoma associated antigen (MCSP) [28] was a gift of Dr. Ralf Reisfeld (Scripps Clinic, La Jolla, CA). Fab fragments of AHN-16 were prepared by using immobilized papain according to the manufacturer's instruction (Pierce, Rockford, IL), and their purity was confirmed by SDS-PAGE analysis.

### Cell adhesion assays

Cell adhesion assays to immobilized mAbs were performed as described previously [29]. Briefly, 96-well plates were coated with 50 ul of goat anti-mouse IgG Fc (Organon Teknika Corp., Durham, NC) overnight at 37°C and then blocked with PBS containing 2 mg/ml BSA for 3 h at 37°C. The purified mAbs were diluted in PBS, dispersed into each well at 1 ug/ml in 50 ul, incubated for 3 to 4 h at 37°C, and then washed 2 times with PBS. Subconfluent A375SM cells that had been radiolabeled overnight with ^3^H-thymidine (^3^H-TdR, specific activity 6.7 Ci/mmol; NEN Research Products, Boston, MA), were harvested by rinsing with PBS containing 1 mM EDTA, washed two times with EMEM/BSA (EMEM containing 2 mg/ml BSA and 15 mM Hepes, pH 7.2), and adjusted to a concentration of 10^5 ^cells/ml in the same medium. When cell adhesion assays were carried out in the presence of 10 mM EDTA or EGTA, cells were resuspended in EMEM/BSA containing 10 mM EDTA or EGTA. An aliquot of 100 ul of the cell suspension was dispersed into each well and incubated for 60 min at 37°C. For inhibiting cell adhesion to the immobilized AHN-16, cells were preincubated with Fab fragments of AHN-16 or of normal mouse IgG for 20 min at room temperature. Cell adhesion assays were carried out in the continuous presence of these Fab fragments for 60 min at 37°C. The assays were terminated by aspirating loosely bound and unbound cells from the wells. Bound radioactivity was determined in a liquid scintillation counter (Beckman Model 3801 Liquid Scintillation Counter).

### Flow cytometry

Cells were harvested as described above, and washed 3 times with RPMI 1640 containing 1% heat-inactivated goat serum, 20 mM HEPES, and 0.02% NaN_3_(FACS buffer). Cells were resuspended in the FACS buffer at a concentration of 10^5 ^cells/ml with 10 ug/ml normal goat IgG for 30 min at 4°C and then washed 3 times with the FACS buffer. An aliquot of 1 ml of cell suspension was incubated with 5 ug/ml of mAbs for 30 min at 4°C with mixing every 5 min. Cells were washed 3 times with the FACS buffer and then incubated with FITC conjugated goat anti-mouse IgG (final dilution of 1: 500) at 4°C for 40 min with mixing every 5 min. The binding of the primary and the secondary mAbs were maximal under these experimental conditions. Cells were washed 3 times with the FACS buffer and resuspended in 200 ul of PBS containing 2% formaldehyde. Cells were analyzed on a FACS Star (Becton-Dickinson, Mt. View, CA).

### Protein kinase assay

Immunoprecipitation was performed as previously described with minor modifications [30]. Briefly, 2 × 10^7 ^cells were suspended in 1.1 ml of Brij solubilization buffer [20 mM Tris-HCl, pH 8.2, 150 mM NaCl, 1 mM PMSF, 2 mM MgCl_2_, 0.02% NaN_3_, and 1.0% Brij 58 (Pierce)], and incubated on ice for 15 min. The suspensions were then centrifuged at 9,800 × g for 15 min at 4°C. The resulting supernatants were used for immunoprecipitation or analyzed by SDS-PAGE directly.

For immunoprecipitations, 1 ml of 10% Staphylococcusaureus (Pansorbin A, Calbiochem, La Jolla, CA) was mixed with 100 μl of rabbit anti-mouse IgG^H+L ^(Organon Teknika, Westchester, PA), and the mixture was incubated at 4°C for 1 hr. One ml of buffer A (1 mg/ml BSA, 0.05% NP-40, 20 mM Tris-HCl, pH 7.6, 100 mM NaCl, 1 mM EDTA, 0.05% NaN_3_) was added and the Pansorbin A-antibody complex was recovered by centrifuging at 2000 × g for 10 min at 4°C and resuspended in 390 μl of buffer A and 40 μl of ascites containing the indicated monoclonal antibody, or normal mouse serum (NMS) was added, and the mixtures were incubated for 2 hr at 4°C. The Pansorbin A-antibody complex was recovered by centrifuging at 2000 × g for 10 min at 4°C, washed once with 1 ml of buffer A, and resuspended in 1 ml of Brij-SA buffer (1 mg/ml BSA, 0.05% Brij 58, 20 mM Tris-HCl, pH 7.6, 100 mM NaCl, 1 mM EDTA, 0.05% NaN_3_). Cell proteins were then immunoprecipitated in reaction mixtures containing the cell extract, Pansorbin A-bound antibody complex, 20 mM Tris-HCl, pH 8.2, 100 mM NaCl, 0.5% Brij 58, 1 mM EDTA, 0.125 mg/ml gelatin, and 1 mM PMSF in a total volume of approximately 350 μl in 10 × 75 mm glass tubes. After 1 hr at 4°C, the mixture was washed three times with Brij wash buffer (20 mM Tris-HCl, pH 8.2, 150 mM NaCl, 1 mg/ml BSA, 0.5% Brij 58, 2 mM MgCl_2_, 0.125 mg/ml gelatin, 1 mM PMSF, and 0.02% NaN_3_) and then once with NaCl-HEPES (145 mM NaCl, 20 mM HEPES, pH 7.3). Immunoprecipitates were suspended in 30 μl of NaCl-HEPES. Thirty ul of labeling buffer (0.1% Brij 58, 6 mM MnCl_2_, 40 mM MgCl_2_, 200 uM Na_3_VO_4_, 200 uM Na_2_MoO_4_, and 10 μCi of [γ^-32^P]ATP) was then added, and the mixtures were incubated for 10 min at 23°C. The reaction was stopped by adding 4 × Laemmli sample buffer, incubated at 100°C for 2 min, and analyzed by SDS-PAGE [31]. Molecular weight standards were purchased from Sigma. Gel slabs were stained, dried, and subjected to autoradiography by using Dupont Cronex film.

### Phosphoamino acid analysis

Phosphoamino acid analyses were performed as previously described [32,33]. Briefly, radiolabeled proteins resolved by SDS-PAGE were transblotted onto Immobilon-P (PVDF) paper (Millipore Corp, Bedford, MA), localized by autoradiography, and excised. The proteins on the PVDF were then hydrolyzed in vacuo in constantly boiling 6 M HCl for 2 hr at 110°C. The HCl was removed by evaporation using a SpeedVac (Savant Instruments). The dried, partially hydrolyzed samples were then dissolved in water containing phosphoserine (P-S), phosphothreonine (P-T), and phosphotyrosine (P-Y) (Sigma) each at 1 mg/ml. The samples were then spotted on aluminum sheets precoated with silica gel (0.2 mm layer thickness, 11 cm height, Merck Laboratories, EM Science, Gibbstown, NJ) and resolved by one-dimensional thin layer chromatography in ethanol: 25% NH_4_OH (3.5:1.6). The chromatography cycle was repeated three times to achieve optimal separation. Between cycles, plates were air dried followed by chromatography in the same solvent. The radiolabeled phosphoamino acids were detected by autoradiography using X-Omat AR film.

## Results

### Human melanoma cells adhere to immobilized CD63 mAbs

**Figure 1:**
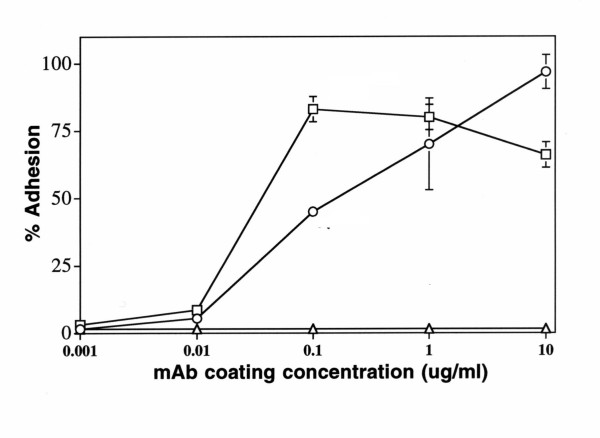
Concentration dependence of A375SM melanoma cell adhesion to immobilized CD63 mAb. Plates were coated with various concentrations of the CD63 mAb (AHN-16) (open circles), the anti-MCSP mAb 9.2.27 (open squares), or normal mouse IgG (open triangles) as described in Materials and Methods. Cell adhesion assays were performed for 30 min. Results are shown as percent adhesion +/- SEM. A duplicate experiment gave similar results.

To establish a simple assay of CD63 signaling in melanoma cells, we tested several CD63 mAbs including AHN-16, AHN-16.1, AHN-16.2, AHN-16.3, and AHN-16.5 for their ability to promote A375SM human melanoma cell adhesion as previously described [29]. CD63 expression on these cells was confirmed using flow cytometry with five CD63 mAbs as described in the Methods (data not shown). Each of the mAbs was immobilized on plastic microtiter plates as described in the Materials and Methods. Cell adhesion assays were carried out for 30 min. At coating concentrations of 1 ug/ml, each of the CD63 mAbs promoted melanoma cell adhesion (Table 1). In contrast, the control antibodies normal mouse IgG (IgG) and the unclustered mAb U131 did not. As expected, the anti-MCSP mAb 9.2.27 promoted high levels of cell adhesion, consistent with our previous results [29]. AHN-16 promoted cell adhesion in a concentration-dependent manner (Fig 1). As expected [29], an anti-melanoma associated proteoglycan mAb (9.2.27) also promoted cell adhesion in a concentration-dependent manner (Fig 1). Normal mouse IgG failed to promote melanoma cell adhesion (Fig 1).

**Table 1:** Human melanoma cell adhesion to immobilized CD63 mAbs

mAb^a^	Specificity	Cell adhesion (%)^b^
AHN-16	CD63	59
AHN-16.1	CD63	29
AHN-16.2	CD63	55
AHN-16.3	CD63	63
AHN-16.5	CD63	29
U131	not clustered	3
IgG^c^	N/A	4
9.2.27	MCSP	88

a. Each mAb was coated on plates at a concentration of 1 ug/ml.

b. Assays of adhesion of A375SM melanoma cells to mAbs immobilized on plastic were performed for 30 min. as described in the text. Cell adhesion is expressed as the percent of the total number of cells added. Values are the means of four replicates that agreed within 10 %. A duplicate experiment gave similar results. c. Normal mouse IgG

**Figure 2:**
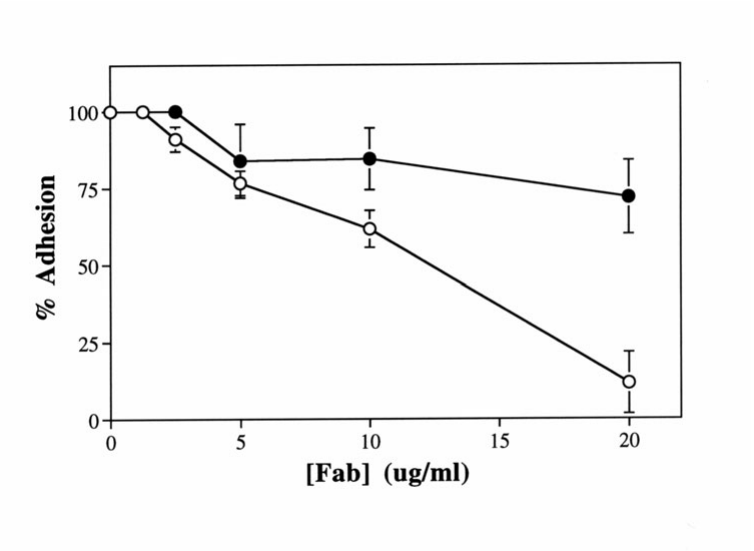
Melanoma cell adhesion to immobilized CD63 mAb was inhibited by Fab fragments. Plates were coated with 0.1 ug/ml of the CD63 mAb AHN-16 as described in Materials and Methods. Cells were preincubated with various concentrations of Fab fragments of AHN-16 (open circles) or of normal mouse IgG (closed circles) for 20 min before adding the cells to the plates for the cell adhesion assays. Cell adhesion assays were performed for 30 min. Results are shown as percent adhesion +/- SEM. A duplicate experiment gave similar results.

In order to confirm that the cell adhesion to immobilized AHN-16 was due to reactivity with the CD63 epitope, Fab fragments of AHN-16 and normal mouse IgG were prepared as described in the Materials and Methods, and tested for their ability to inhibit melanoma cell adhesion to immobilized AHN-16. Fab fragments of AHN-16, but not of normal mouse IgG, effectively inhibited cell adhesion to AHN-16 in a concentration dependent manner (Fig 2). At the highest concentration of AHN-16 Fab fragment (20 ug/ml), cell adhesion was inhibited by 90%. These results confirmed that cell adhesion to immobilized AHN-16 was dependent on the antigen binding site.

### Human melanoma cell adhesion to immobilized CD63 mAb is inhibited by chelerythrine

**Figure 3:**
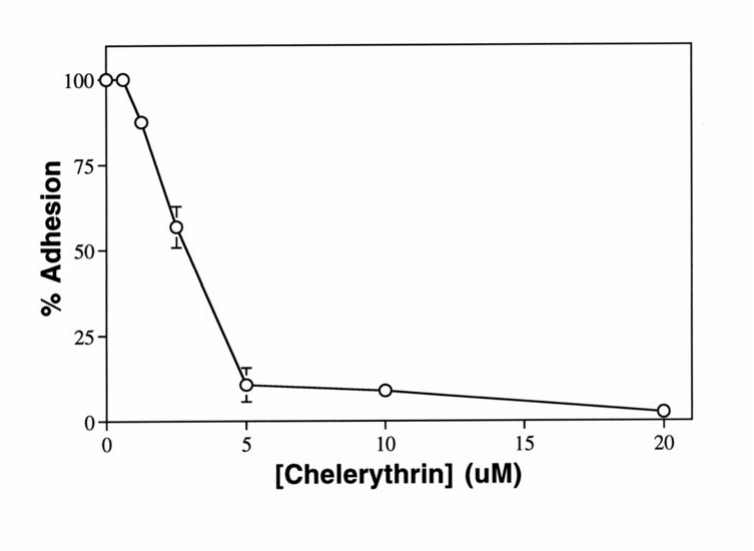
Chelerythrine inhibited cell adhesion to immobilized CD63 mAb. Plates were coated with 0.1 ug/ml of the CD63 mAb AHN-16 or the anti-MCSP mAb 9.2.27 as described in Materials and Methods. Cells were preincubated with various concentrations of chelerythrine for 20 min before adding the cells to the plates for the cell adhesion assays. Cell adhesion assays were performed for 30 min. Results are shown as percent adhesion +/- SEM. A duplicate experiment gave similar results.

In order to study the signaling pathways by which CD63 may transmit signals, cell adhesion assays were carried out in the presence of chelerythrine, a potent and specific inhibitor of protein kinase C [34]. Melanoma cells were preincubated with various concentrations of chelerythrine or genistein, a selective tyrosine protein kinase inhibitor, for 20 min at 37°C prior to cell adhesion assays. Chelerythrine inhibited melanoma cell adhesion to immobilized AHN-16 in a concentration dependent manner with an IC_50 _of approximately 2.5 uM (Fig 3). Chelerythrine did not alter surface expression of CD63 as determined by flow cytometry (not shown). In contrast, genistein did not affect cell adhesion to immobilized AHN-16 under these conditions at concentrations up to 50 ug/ml (not shown). However, cell adhesion to mAb 9.2.27 was totally resistant to both chemicals (not shown). These results suggested that protein kinase C plays a role in cell adhesion to immobilized CD63 mAb.

### Human melanoma cell adhesion to immobilized CD63 mAb requires external Ca^2+^

**Figure 4:**
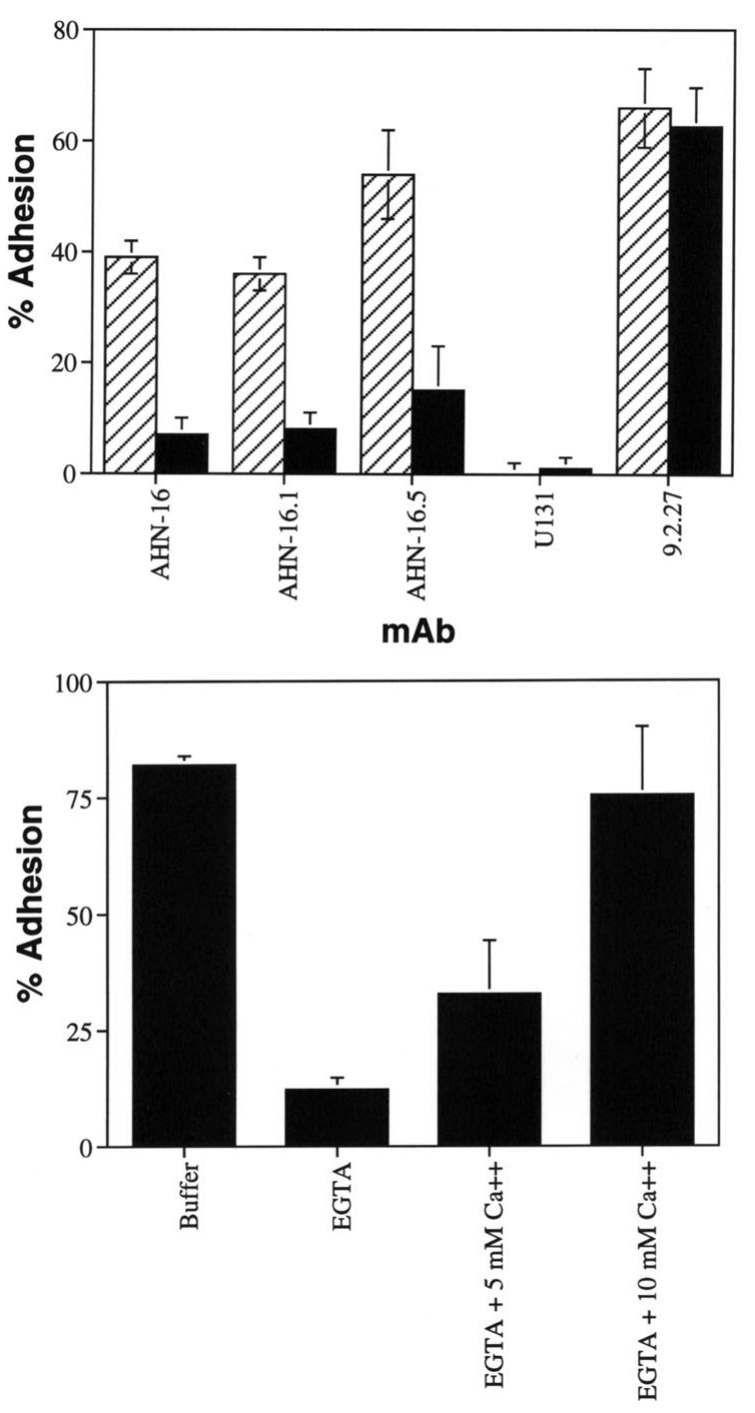
EGTA inhibited cell adhesion to immobilized CD63 mAb. Top panel: plates were coated with 0.1 ug/ml of the indicated mAb and cell adhesion assays were carried out in the absence (hatched bars) or presence (solid bars) of 10 mM EGTA for 30 min as described in the Materials and Methods. CD63 mAbs: AHN-16, AHN-16.1, AHN-16.5; unclustered control mAb: U131, anti-MCSP mAb: 9.2.27. Bottom panel; plates were coated with 0.1 ug/ml of the CD63 mAb AHN-16 as described in the Materials and Methods, and cell adhesion assays were performed in either 10 mM EGTA, 10 mM EGTA plus 5 mM Ca^2+^, or 10 mM EGTA plus 10 mM Ca^2+ ^for 30 min as indicated. Results are shown as percent adhesion +/- SEM. A duplicate experiment gave similar results.

We next examined the effects of extracellular Ca^2+ ^on melanoma cell adhesion to immobilized CD63 mAbs. Human melanoma cell adhesion to three different immobilized CD63 mAbs was almost totally inhibited by EGTA, indicating that external Ca^2+ ^plays an important role in human melanoma cell adhesion to the mAbs (Fig 4, top). Cells did not adhere to immobilized CD63 mAbs in the absence of calcium, suggesting that this adhesion is an active process. In contrast, cell adhesion to immobilized mAb 9.2.27 was totally resistant to EGTA (Fig 4A, top). EGTA itself was not inhibitory since cell adhesion to AHN-16 was similar to control cell adhesion levels when a normal Ca^2+ ^level was generated by adding 10 mM Ca^2+ ^to the cells in EGTA (Fig 4B, bottom). EGTA did not interfere with the interaction between human melanoma cells and AHN-16, since AHN-16 bound the cell surface normally as assessed by flow cytometery (not shown).

In some cells, for example human neutrophils, the binding of a stimulus to its receptor on the cell surface results in the activation of a signal transduction system that is transient, and whose function is dependent on extracellular Ca^2+ ^[35,36]. When calcium is absent during a critical period following ligand-receptor coupling, the subsequent addition of calcium does not result in cell activation, and the cells may be partially desensitized to subsequent stimulation in the presence of Ca^2+ ^[35,36]. The role of extracellular Ca^2+ ^was therefore further evaluated. When calcium (10 mM) was added to the cells in medium with EGTA immediately after adding them to the plate, adhesion was normal. However, if the cells were allowed to interact with the immobilized CD63 mAbs for 20 min before repleting the calcium, adhesion was not restored by the calcium (not shown).

### Identification of protein kinase activity associated with CD63

**Figure 5:**
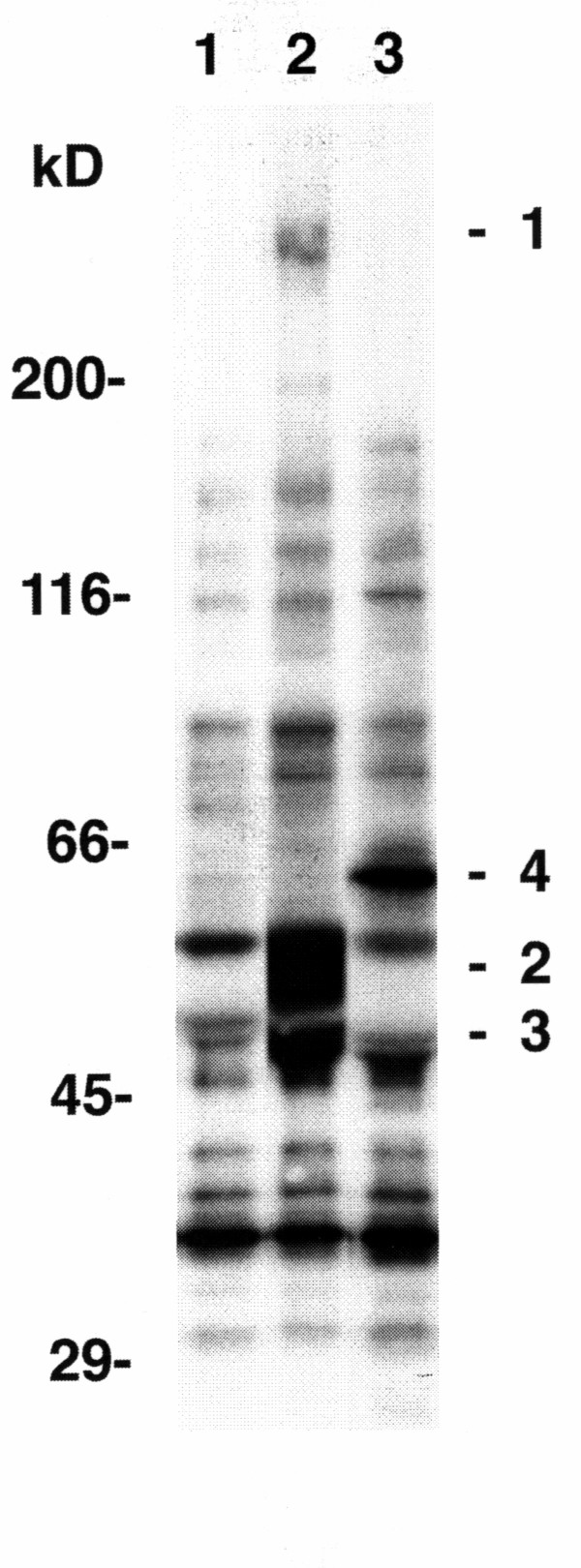
Co-immunoprecipitation of protein kinase activity with CD63. Panel A, Cells were solubilized in Brij solubilization buffer and immunoprecipitated with NMS (lane 1), the CD63 mAb AHN-16 (lane 2), or the anti-MCSP mAb 9.2.27 (lane 3), and the immunoprecipitates were incubated with [γ^-32^P]ATP as described in the text. The resulting phosphoproteins were resolved by SDS-PAGE and visualized by autoradiography as described in the text. Three phosphoproteins were reproducibly identified in CD63 mAb immunoprecipitates (labeled 1–3 in lane 2). One phosphoprotein was reproducibly identified in immunoprecipitates using the anti-MCSP mAb 9.2.27 (labeled 4 in lane 3). These four phosphoproteins were not seen in the immunoprecipitate using NMS (lane 1). Proteins used as molecular weight standards were: myosin heavy chain, 200,000; Escherichia coli β-galactosidase, 116,000; bovine serum albumin, 66,000; ovalbumin, 45,000; and carbonic anhydrase, 29,000.

**Figure 6:**
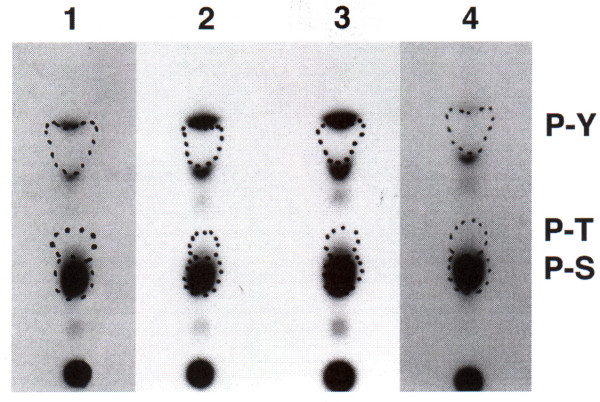
Phosphoamino acid analysis of proteins co-precipitated with the CD63 mAb AHN-16 and the anti-MCSP mAb 9.2.27, and labeled in an in vitro kinase assay. Cells were solubilized, immunoprecipitated, labeled with [γ^32^P-]ATP, and the resulting phosphoproteins were resolved by SDS-PAGE as in Fig 5. Phosphoproteins were transferred to Immobilon by electroblotting, visualized by autoradiography, excised, and subjected to acid hydrolysis. The resultant phosphoamino acids were resolved by thin layer chromatography and visualized by autoradiography as described in the text. The positions of migration of authentic phosphoserine (P-S), phosphothreonine (P-T), and phosphotyrosine (P-Y) are indicated by dotted lines. A duplicate experiment gave similar results. The lane numbers correspond to the phosphoproteins numbered in figure 5.

Since protein phosphorylation appears to be involved in signaling by CD63 in A375SM cells, we questioned whether protein kinase activity could be associated with CD63 in A375SM cells as has been observed in neutrophils [15]. A375SM melanoma cells were solubilized in a buffer containing Brij 58 as described in the Methods, and the solubilized material was immunoprecipitated by CD63 mAbs. When [γ^-32^P]ATP was added to the material immunoprecipitated by the CD63 mAb AHN-16 (Fig 5, lane 2) or the CD63 mAb AHN-16.1 (not shown), ^32^P was reproducibly incorporated into three distinct proteins of ~208 to 236-kD, 53 to 58-kD, and 46 to 50-kD (labeled 1–3), that were not present when material was immunoprecipitated by NMS (Fig 5, lane 1). In contrast, ^32^P was reproducibly incorporated into a ~66-kD protein (labeled 4) in material immunoprecipitated by mAb 9.2.27 (Fig 5, lane 3). Each of these four individual proteins were examined for phosphoamino acid content as described in the Methods. The majority of radiolabel in each of these four proteins was present on serine residues (Fig 6), demonstrating the presence of serine kinase activity.

## Discussion

Immunohistochemistry studies have characterized CD63 as the stage-specific melanoma-associated antigen ME491 [6-8]. Over expression of CD63 partially suppressed malignant phenotypes of H-ras-transformed fibroblasts in vivo[19], and transfection of melanoma cell lines also suggested that CD63 can regulate melanoma cell function [20,21]. The current study also demonstrates that CD63 can transduce signals in melanoma cells and alter melanoma cell behavior. In addition, this study demonstrates that CD63 is capable of transducing signals in melanoma cells that requires extracellular Ca^2+ ^and is inhibited by the protein kinase C inhibitor chelerythrine. Signaling by tetraspans, including the induction of calcium-dependent cell-cell adhesion, has been reported in other systems [37-39].

To examine potential signal transducing pathways through which CD63 may act, an in vitro assay measuring melanoma cell adhesion to immobilized mAbs was used. We previously utilized this method for characterizing molecular mechanisms of signal transduction from proteoglycan as well as integrin receptors in melanoma cells [29,40]. Melanoma cells specifically adhered to immobilized CD63 mAb, and the adhesion was inhibited by chelerythrine but not by genistein, suggesting that signaling pathways involving protein kinase C activity, but not genistein-sensitive tyrosine kinases, play a role in the stimulation by CD63. The inhibitory activity of chelerythrine was not due to cytotoxicity, since the chemicals did not affect cell adhesion to immobilized mAb 9.2.27. The IC_50 _value of chelerythrine is close to the value that inhibits the activity of protein kinase C in vitro[34], supporting the conclusion that the inhibitory effect of chelerythrine on cell adhesion to immobilized CD63 mAb is of a specific nature.

CD63 may interact with different signaling molecules in different cell types. In human neutrophils, tyrosine protein kinase activity has been reported to be associated with CD63; most of this tyrosine kinase activity was found to be due to the src family tyrosine kinases lyn and hck which coimmunoprecipitated with CD63 in neutrophils, suggesting that these kinases may play a role in signaling via CD63 [15]. In the current study, we found serine protein kinase activity, but could not detect tyrosine kinase activity, in immunoprecipitates using CD63 mAbs. Recent studies have reported that CD63 in platelets modulates spreading and tyrosine kinase activity [41]. Thus, these results indicate that the interaction between CD63 and these kinases is dependent upon the cell type. These data also support a role for protein kinase C in CD63 signaling in melanoma cells. CD63 has also been found to associate with the β2 integrin CD18 in neutrophils and the β1 integrin in several other cells, including melanoma cells, and this may also play a role in signaling by CD63 [15,42-45]

Melanoma cell adhesion to immobilized CD63 mAb required extracellular calcium, indicating that extracellular Ca^2+ ^plays an important role in signaling by CD63 in these cells. Addition of calcium to cells 20 min after interaction of cells with immobilized CD63 mAb resulted in no adhesion. Thus, the interaction of melanoma cells expressing CD63 to immobilized CD63 mAbs resulted in the generation of a transient signal that required extracellular calcium to effect melanoma cell adhesion. These observations are similar to those reported with human neutrophils where ligation of CD63 results in a transient activation signal that requires extracellular calcium [15].

## Conclusion

The current study demonstrates that CD63 is capable of transducing signals in melanoma cells that requires extracellular Ca^2+ ^and is inhibited by the protein kinase C inhibitor chelerythrine, and that CD63 is associated with serine protein kinase activity in melanoma cells. This associated protein kinase activity may play a role in the mechanism whereby CD63 exerts its previously reported effects on melanoma cell function [20,21].

## Abbreviations

Abbreviations used in this paper: EMEM, Eagle's minimum essential medium; NMS, normal mouse serum; FACS buffer, RPMI 1640 containing 1% heat-inactivated goat serum, 20 mM HEPES, and 0.02% NaN_3_; NaCl-HEPES, 145 mM NaCl, 20 mM HEPES, pH 7.3; Brij solubilization buffer, (20 mM Tris-HCl, pH 8.2, 150 mM NaCl, 1 mM PMSF, 2 mM MgCl_2_, 0.02% NaN_3_, and 1.0% Brij 58); Brij wash buffer, (20 mM Tris-HCl, pH 8.2, 150 mM NaCl, 1 mg/ml BSA, 0.5% Brij 58, 2 mM MgCl_2_, 0.125 mg/ml gelatin, 1 mM PMSF, and 0.02% NaN_3_); labeling buffer, (0.1% Brij 58, 6 mM MnCl_2_, 40 mM MgCl_2_, 200 uM Na_3_VO_4_, 200 uM Na_2_MoO_4_, and 10 μCi of [γ^-32^P]ATP); buffer A, (1 mg/ml BSA, 0.05% NP-40, 20 mM Tris-HCl, pH 7.6, 100 mM NaCl, 1 mM EDTA, 0.05% NaN_3_); buffer B, (PBS, pH 7.4, 0.2% BSA, 0.05% NaN_3_); Brij SA buffer, (1 mg/ml BSA, 0.05% Brij 58, 20 mM Tris-HCl, pH 7.6, 100 mM NaCl, 1 mM EDTA, 0.05% NaN_3_, 1 mM Na_3_VO_4_, and 1 mM Na_2_MoO_4_)

## Competing interests

The author(s) declare that they have no competing interests.

## Authors' contributions

GI helped conceive the study, carried out the adhesion assays and antibody purification, participated in experimental design and data analysis, and helped draft the manuscript

APNS helped conceive the study, design experiments, analyze data, and draft the manuscript

JBM helped analyze data and draft the manuscript

KMS helped conceive the study, design experiments, analyze data, and draft the manuscript
